# Treasurer’s Report of the American Veterinary Medical Association1Presented at the Thirty-eighth Annual Meeting.

**Published:** 1901-12

**Authors:** 


Secretary.

---

### TREASURER'S REPORT OF THE AMERICAN VETERINARY MEDICAL ASSOCIATION.[1]

1900. RECEIPTS.

| | | |
|---|---|---|
| Balance at last report . . . . . . . | $770 28 | |
| Received from Dr. S. Stewart, Secretary, September 22, 1900, to September 2, 1901 . . . . | 2215 18 | |
| | | $2985 46 |

DISBURSEMENTS.

| | | |
|---|---|---|
| Dr. Stewart, Secretary, salary, expenses for postage, express, lettering certificates . . . . . . | $323 80 | |
| Mr. T. E. Crossman, stenographer, Detroit meeting | 112 50 | |
| Allowance for annual banquet and balance of Secretary's expenses . . . . . . . . | 72 22 | |
| Army Legislative Committee work . . . . . | 279 12 | |
| Lane Printing Co., Kansas City, Kansas, printing letter-heads, etc. . . . . . . . . | 175 88 | |
| Pioneer Press Co., 400 copies Proceedings, and mailing and express charges on same . . . . . | 439 25 | |
| State Secretaries' expenses . . . . . . | 231 68 | |
| Treasurer's expenses . . . . . . . . | 12 40 | |
| Dr. W. L. Williams, Librarian, expenses . . . | 9 61 | |
| American Surety Co., of New York, payment of premium on Treasurer's bond . . . . . | 10 00 | |
| | | 1666 46 |
| Balance on hand . . . . . . . . . | | $1319 00 |

[1] Presented at the Thirty-eighth Annual Meeting.

